# Design and implementation of an AAPM volunteer database for advancing global medical physics initiatives

**DOI:** 10.1002/acm2.70511

**Published:** 2026-02-19

**Authors:** Teh Lin, Minsun Kim, Courtney Morrison, Jiahan Zhang, Kevin Little, Yan‐Hong Xin, Michael Woodward, Peter Sandwall, Eun Young Han

**Affiliations:** ^1^ Department of Radiation Oncology Fox Chase Cancer Center Philadelphia Pennsylvania USA; ^2^ Department of Radiation Oncology University of Texas Southwestern Medical Center Dallas Texas USA; ^3^ Department of Radiology Rush University Medical Center Chicago Illinois USA; ^4^ Department of Radiation Oncology Icahn School of Medicine at Mount Sinai New York New York USA; ^5^ Department of Radiology The Ohio State University Columbus Ohio USA; ^6^ Information Technology Department American Association of Physicists in Medicine Alexandria Virginia USA; ^7^ Department of Radiation Oncology OhioHealth Mansfield Ohio USA; ^8^ Department of Radiation Physics The University of Texas MD Anderson Cancer Center Houston Texas USA

**Keywords:** global oncology, informatics, LMIC, medical physics volunteerism

## Abstract

**Purpose:**

The purpose of this study is to assess American Association of Physicists in Medicine (AAPM) members' availability to volunteer, share their expertise, and promote global collaboration in medical physics by partnering with international colleagues who seek support in strengthening their medical physics practice.

**Methods:**

The questionnaire was administered to AAPM members to assess their willingness to volunteer, preferred engagement modalities, and availability for different types of volunteer activities. The questionnaire was integrated into the AAPM member profile and covered therapy, diagnostic, and nuclear medicine disciplines, capturing participants' visiting preferences and expertise in teaching and training. Respondents were categorized based on demographic factors such as years of experience, expertise areas, and geographic location.

**Results:**

A total of 409 AAPM members completed and submitted 427 responses to the questionnaire (18 members submitted two discipline‐specific sets). The 4% response rate confines this report to exploratory findings rather than predictive conclusions for all AAPM members. Most respondents expressed a preference for short‐term engagements, with 49.2% favoring visits of less than one week. Volunteers reported significant expertise, with a substantial portion having more than 15 years of professional experience. Additionally, respondents demonstrated active participation in international activities, with 57 engaged in AAPM initiatives and over 100 involved in private nonprofit or nongovernmental organizations (NGOs).

**Conclusions:**

This report highlights the breadth of expertise and willingness of AAPM members to collaborate and support education across all specialties, contributing to the advancement of global medical physics. While the volunteer data shows significant experience and expertise, targeted recruitment is needed in certain specialized areas. This initiative offers a scalable and sustainable model for global volunteerism in medical physics, complementing traditional in person training and addressing the needs of low‐ and middle‐income countries (LMICs).

## 1 INTRODUCTION

The overarching goal is to identify and develop strategies that advance the practice of medical physics globally and help address disparities in healthcare.^1^ We also aim to collaborate with international stakeholders—including medical physics organizations and nonprofit groups—to strengthen these efforts. Significant global health work has already been undertaken in radiation oncology,^2^, ^3^, ^4^, ^5^, ^6^ diagnostic radiology,^7^, ^8^, ^9^, ^10^, ^11^, ^12^, ^13^ and nuclear medicine.^14^, ^15^, ^16^, ^17^ Many of these initiatives have focused on education and training for personnel in low‐ and middle‐income countries (LMICs).^9^, ^18^, ^19^, ^20^, ^21^, ^22^, ^23^, ^24^, ^25^, ^26^, ^27^, ^28^, ^29^, ^30^, ^31^ For example, a recent AAPM‐led survey highlighted a major challenge in LMICs: assessing radiology and radiation therapy needs for cancer care. Forty‐one percent of respondents identified insufficient training programs as the primary barrier to adopting advanced technologies such as Ethos and Halcyon (Varian Medical Systems, Palo Alto, CA). Moreover, respondents emphasized that in person training is preferred for gaining hands‐on experience before deploying advanced equipment, ranking it highly among educational needs.^32^, ^33^, ^34^ Although AAPM members—primarily from high‐income countries—possess valuable expertise and resources, many struggle to identify and engage in meaningful international collaboration opportunities.

Given these difficulties, we recognized the need for a volunteer database to maximize its work. We were tasked with creating and maintaining a list of potential volunteers within the AAPM membership who would be willing and able to volunteer their time and effort towards various international activities, such as collaborating with institutions and partners in different world regions to address global needs. Each potential volunteer has their own strengths that would ideally be well matched with volunteer opportunities. Volunteers may have expertise in a particular treatment or imaging modality, extensive involvement in training and education, or experience and contacts in a specific geographic region. Potential volunteers also have varying time and financial resources that may be committed to an opportunity. Understanding a potential volunteer's strengths and constraints allows for the optimal match of the volunteer with available opportunities.

This manuscript serves as a precursor to future efforts by the authors, aiming to assess whether existing AAPM initiatives align with member interests and global needs, and to identify areas where additional focus or resources may be warranted. With these goals in mind, the authors developed a questionnaire for potential volunteers that captures these data. The data have been extracted from AAPM members’ profile respectively, providing a persistent database that can be used by groups to match volunteers to serving opportunities. This work provides an analysis of the first batch of questionnaire respondents, providing an overview of potential volunteer resources.

## 2 MATERIALS AND METHODS

First, three discipline‐specific questionnaires were developed, covering the fields of therapy, diagnostic, and nuclear medicine physics. The questionnaires included the major categories of technical expertise, specific areas of interest for training or teaching, involvement in international activities, visiting preference, funding requirement, and preferred duration of various volunteer activities. We consulted experts in each discipline and referenced taxonomy from a medical physics journal to compile a comprehensive list of specific areas of interest. Second, the authors collaborated with AAPM's Information Technology (IT) department to refine the questionnaires and convert them to web versions. As a pilot study, the web versions were circulated to a small group (< 100) to test connectivity and provide feedback. Third, after reviewing feedback and incorporating changes, the final version was deployed to all AAPM members in March 2023 in the form of a member profile update.

**FIGURE 1:**
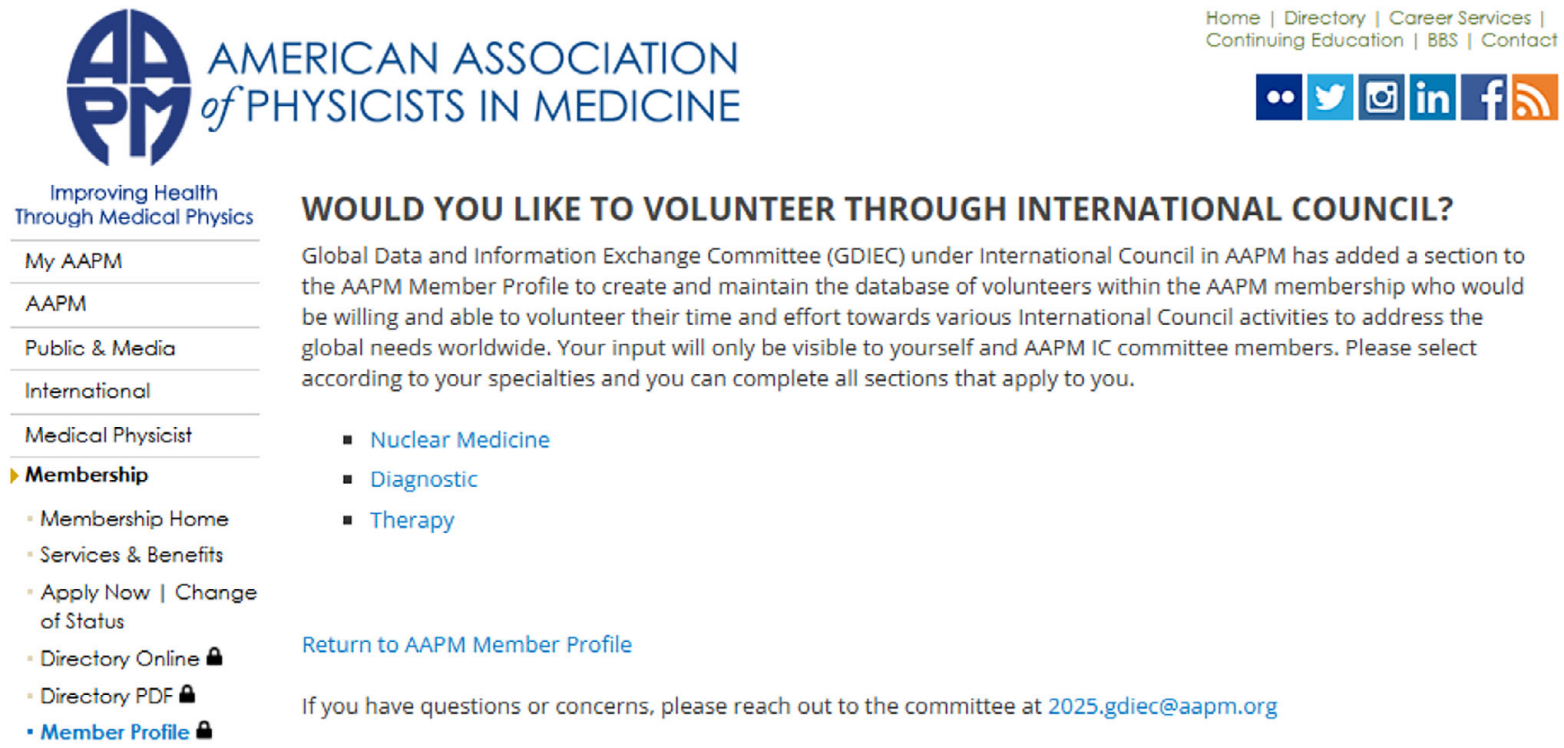
Screenshot of the questionnaire user interface at AAPM.org member profile.

AAPM members were made aware of the profile update through the AAPM newsletter, local chapters, medical physics listserv, and social media platforms. All responses completed by December 2023 were analyzed and included in this work. Figure 1 shows a screenshot of the questionnaire user interface.

In January 2024, AAPM revised its membership structure; the classes reported in this work reflect the updated membership structure. The questionnaire allowed both prespecified options (drop‐down menu) and free‐text entries. The full questionnaire, including the exact wording of each question and the complete set of available response options, has been provided as Supplementary Material. Readers can also find the online questionnaire at the web address: www.aapm.org/memb/profile/gdiec_volunteer.asp

## 3 RESULTS

A total of 409 physicists across all AAPM membership classes responded, and 18 indicated two disciplines (both nuclear medicine and diagnostic), resulting in 427 responses. Note that members were able to complete multiple sub‐specialty questionnaires. Of the 409 members submitting questionnaires, 81.1% were from full members (332), 6.4% were from general members (26), 8.1% were from associate members (33), and 4.4% were from affiliate members (18). Among the 427 responses, 302 members completed the therapy questionnaire, representing 70.7% of the total 427 responses, while 88 (20.6%) completed the diagnostic questionnaire and 37 (8.7%) completed the nuclear medicine questionnaire.

### 3.1 Visiting preferences

Regarding geographical volunteering preferences, a total of 2,144 entries were collected, as participants were permitted to select multiple regions. The responses were relatively evenly distributed across all regions, including North America (15%), South America (15%), Asia (18%), the Middle East (12%), Africa (12%), Europe (16%), and Oceania (12%), with all regions falling within a narrow range of 12–18%. This broad distribution reflects a global willingness among AAPM members to participate in international initiatives.

**FIGURE 2:**
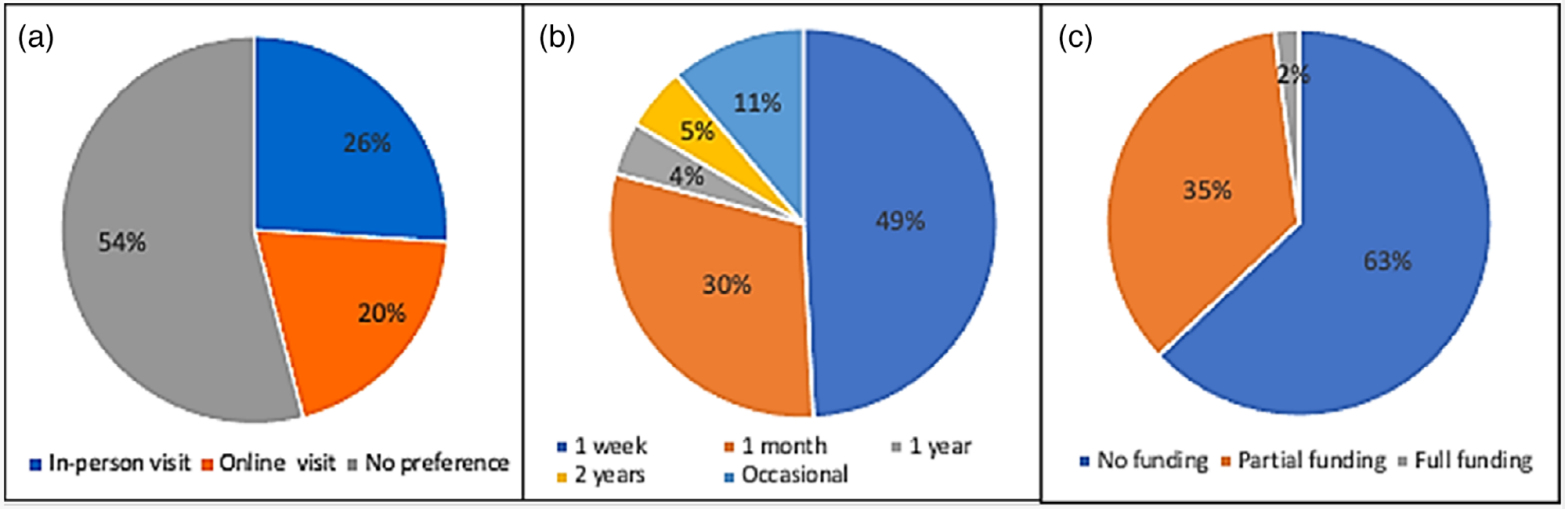
Visiting preferences. (a) preference for online versus in person visits (*n* = 398). Majority of the members (54%) have no preference, (b) preferred duration of volunteer visits (*n* = 364). 49% of members prefer 1 week of volunteering service, and (c) primary funding sources for participation (*n* = 270). Majority of the members (63%) do not have funding.

Figure 2 presents the visiting logistics preferences of AAPM members who responded to the questionnaires. It includes information on the preferred method of visit (online vs. in person) (Figure 2(a)), the preferred duration of international volunteering (Figure 2(b)), and the availability of funding for international travel (Figure 2(c)). In particular, online visits refer to virtual lectures and training delivered via video conference platforms. More than half of respondents (54%, *n* = 398) expressed no preference between online and in person visits. Among 364 respondents, 49.2% preferred visits of less than one week, 30.0% less than one month, 4.4% less than one year, 5.2% less than two years, and 11.2% indicated occasional visits. Figure 2(c) illustrates the funding availability for international travel, with 63% (169 out of 270 total responses) of volunteers having no direct funding source and 35% (94 out of 270 total responses) having partial funding.

The distribution of years of professional experience among 409 respondents showed that 22.9% had less than 5 years of experience, 18.5% had 5–10 years, 16.3% had 10–15 years, and 42.2% had more than 15 years of experience. This indicates that more than half of the volunteers are highly experienced professionals, with a broad representation across career stages.

### 3.2 Specialty areas of training/teaching

**FIGURE 3:**
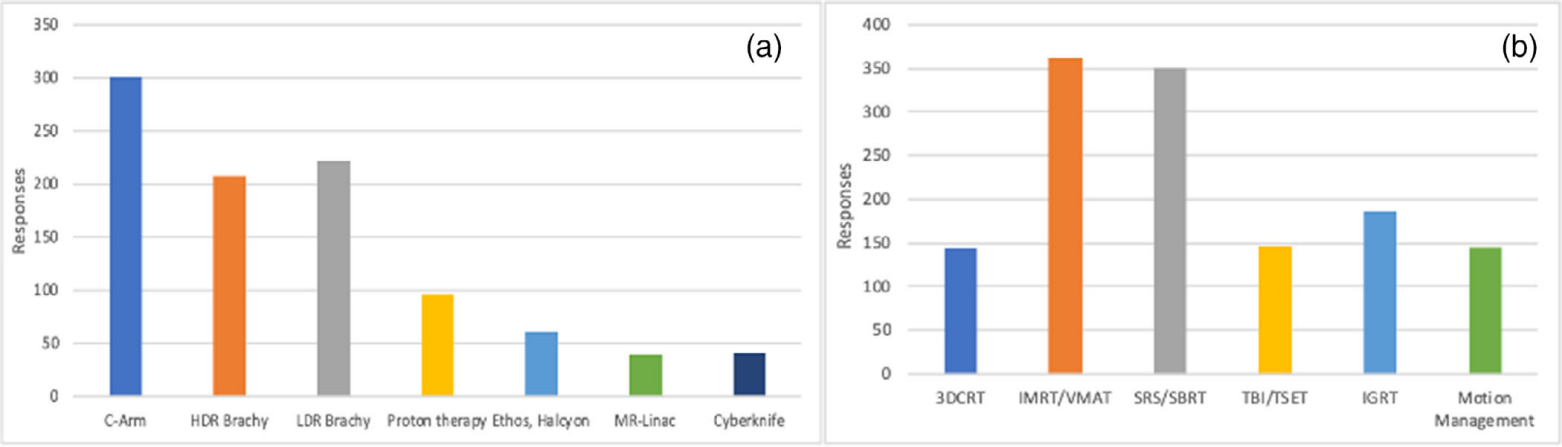
Expertise indicated in a total of 967 responses in (a) radiotherapy machines and (b) therapy specialty areas of training and teaching.

For volunteer expertise questions, 967 entries were collected. Respondents were allowed to select all options that applied. This question set also asked the volunteers to select the specialties in which volunteers were comfortable teaching and providing training. Figure 3(a) highlights the distribution of expertise in radiotherapy machines, and Figure 3(b) outlines the radiotherapy specialties in which volunteers are comfortable providing training. Among the therapy responses, more than 200 people indicated expertise in C‐arm machines and brachytherapy, while about 50 people indicated expertise in newer technologies such as MR‐linacs, Ethos, and Halcyon. More than 350 people said they were comfortable teaching about IMRT/VMAT and SRS/SBRT. Specialty devices such as Gamma Knife reported by only a few respondents were excluded from Figure 3 for simplicity. More volunteers were willing to assist with IMRT/VMAT topics than with 3DCRT. This likely reflects that most AAPM members work in high‐resource settings where IMRT/VMAT are standard practice, making them more comfortable teaching these modalities, while assuming 3DCRT techniques are already well established.

**FIGURE 4:**
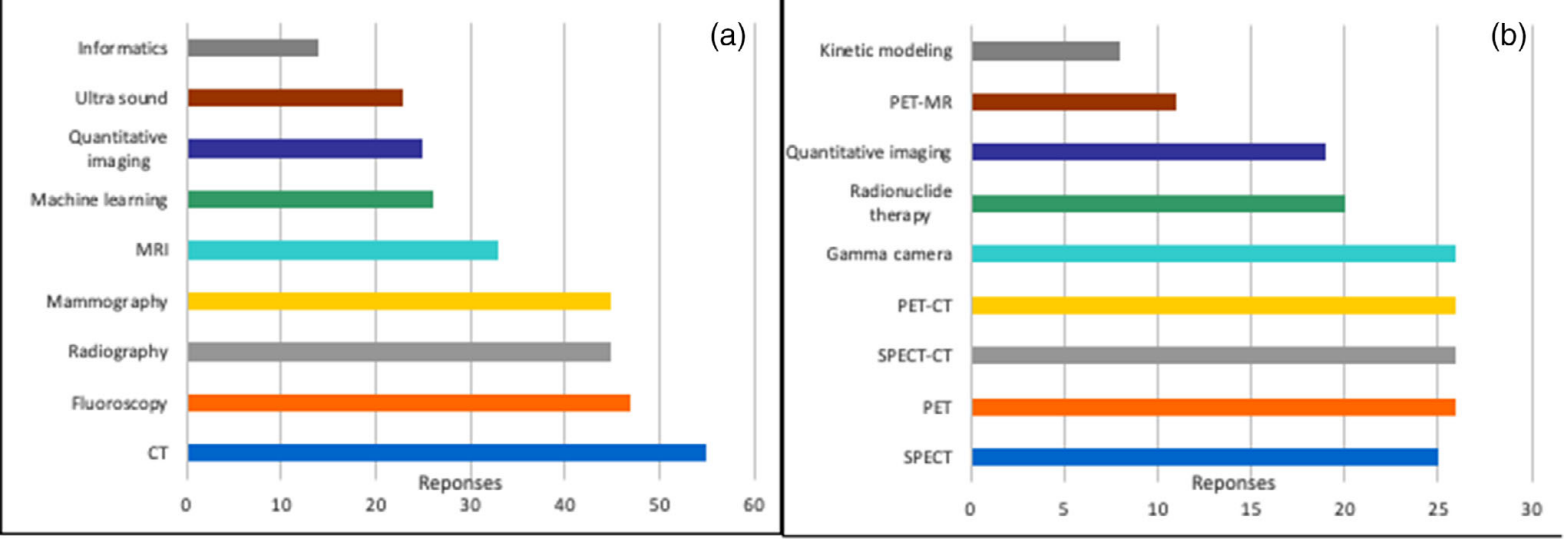
Specialty areas of training/teaching for (a) diagnostic medical physics (a total of 369 responses), and (b) nuclear medicine physics (a total of 132 responses).

Figure 4 depicts the specialty areas in which volunteers were willing to provide training and teaching, focusing on (a) diagnostic medical physics and (b) nuclear medicine physics. Among the diagnostic responses (a total of 369 responses), the areas with the highest number of respondents indicating expertise were CT, fluoroscopy, radiography, and mammography, while SPECT, PET, SPECT‐CT, PET‐CT, and gamma cameras received the highest number of responses on the nuclear medicine questionnaire (a total of 132 responses).

### 3.3 Current involvement of international activities

**FIGURE 5:**
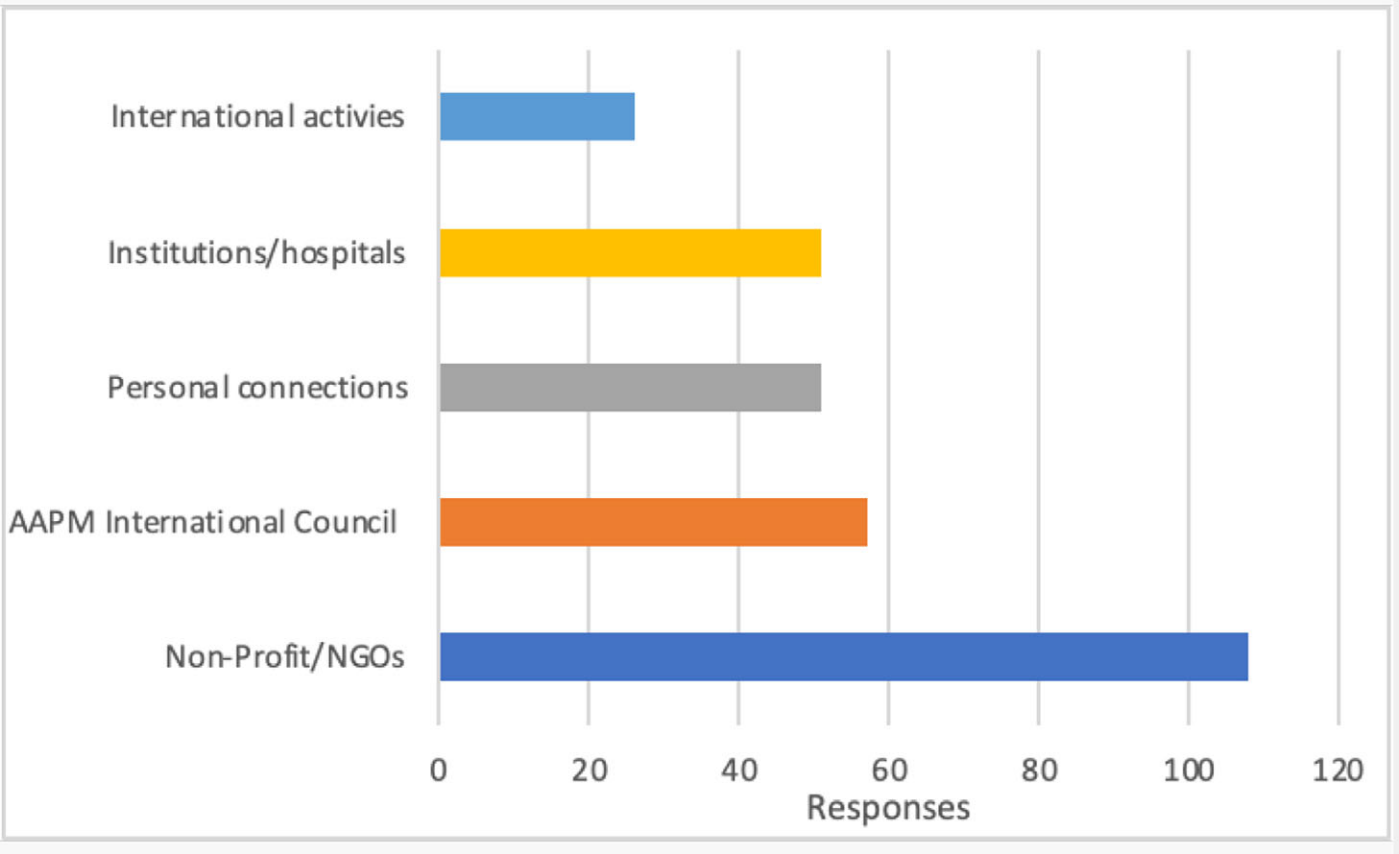
Current involvement in international activities (a total of 293 responses).

For current involvement in international activities, 293 total entries were collected. Note that the categories are not mutually exclusive, and some respondents selected multiple options for their involvement in international activities. These international activities include participation in the following organizations: IAEA—International Atomic Energy Agency, ASTRO—American Society for Radiation Oncology, ESTRO—European Society for Radiotherapy and Oncology, COG—Children's Oncology Group (commonly used in oncology/radiotherapy contexts), IOMP—International Organization for Medical Physics, EFOMP^35^—European Federation of Organizations for Medical Physics, and ICRU—International Commission on Radiation Units and Measurements. Figure 5 presents the number of responses indicating participation in various organizations. The figure highlights that 57 members were already involved through AAPM international committee activities, and more than 100 members were currently engaged with nonprofit or nongovernmental organizations (NGOs).

## 4 DISCUSSION

In early 2023, we launched the first initiative to assess AAPM members’ willingness to contribute their expertise on a global scale. This effort established a comprehensive volunteer database designed to connect members with physicists seeking support worldwide, thereby fostering international collaboration and knowledge exchange. Similar initiatives have recently emerged, such as the EFOMP YOU^35^ online portal, which promotes mentorship and engagement across European member states, and Physics Without Frontiers, which coordinates volunteer scientists to support education and research in the Global South. Medical Physics for World Benefit (MPWB) also engages physicists in capacity‐building efforts internationally. Our work complements these initiatives by providing a dedicated platform for global volunteerism within AAPM, emphasizing scalable, membership‐driven participation.

This effort should also be viewed in the context of broader international initiatives, such as the IAEA's Rays of Hope program, which focuses on strengthening cancer care in LMICs through radiotherapy infrastructure development, regional anchor training centers, and capacity building. Unlike this large‐scale program, the AAPM volunteer network provides a flexible, member‐driven mechanism for targeted expertise sharing and mentorship, offering a complementary pathway to support sustainable capacity building. In parallel, AAPM has also taken steps toward supporting long‐term international engagement through programs such as the International Council Associates Mentorship Program (ICAMP), which provides structured mentorship and financial support for early‐career medical physicists involved in global activities ($2000). Although primarily focused on workforce development, initiatives such as ICAMP demonstrate institutional commitment and offer a potential foundation for continued growth and sustainability of AAPM's global outreach efforts.

The questionnaire implementation occurred in two stages: firstly, a pilot study involving a small group of physicists who were familiar with the goal of this project and could provide direct feedback, and subsequently, the launch of a comprehensive roll‐out to all AAPM members. This sequential approach enabled us to enhance and fine‐tune the questionnaires based on valuable insights and feedback collected during the initial pilot phase. To maximize inclusivity during the full‐scale implementation, various strategies were employed to encourage member participation. This multifaceted approach aimed to ensure broad representation and participation in the questionnaire.

The design and construction of the questionnaires and databases were carried out in close collaboration with the AAPM IT department. Collaboration with the IT team not only provided technical support but also improved the database's usability, ensuring accessibility for a larger group of members. Early coordination to clarify project needs helped prevent resource waste and reduced time‐consuming revisions later in the process. This approach, marked by strategic collaboration and planning, enhanced efficiency and created a more seamless experience for all involved.

To further streamline and enhance the efficiency of the matching process, AAPM member profiles were strategically utilized as the main repositories of volunteer information. This allows members to conveniently indicate and update their areas of expertise, preferences, and availability to volunteer for specific topics at any time. Moreover, essential information for the matching process can be extracted directly from member profiles, minimizing the need to provide duplicate details in the questionnaire and ensuring a smoother, more accessible experience. The IT department also developed a search function to query the volunteer database, enabling efficient identification of volunteers with specific skills and availability.

These findings provide important insight into how volunteer engagement might be structured in practice. The preference for shorter visits likely reflects practical considerations such as limited vacation time, clinical responsibilities, and logistical constraints, highlighting the importance of designing flexible, short‐term volunteer opportunities to maximize participation. Furthermore, the high proportion of volunteers with more than 15 years of professional experience suggests a strong foundation of expertise that can be leveraged to support complex clinical and educational needs in LMICs. At the same time, the presence of early‐ and mid‐career professionals indicates opportunities for sustained engagement and capacity building over time.

This initiative provides valuable insights into the willingness of AAPM members to participate in international volunteerism. Beyond documenting volunteer distributions, our findings highlight the practical impact of this database. Remote engagement offers clear advantages over bringing physicists from LMICs to high‐income institutions for training, including reduced financial burden, improved accessibility, scalability, and the ability to provide continuous support over time. In addition, volunteers themselves benefit from opportunities for global health engagement, professional growth, and cross‐cultural exchange, while LMIC institutions gain access to targeted expertise and sustained mentorship. This initiative is also important to disseminate within AAPM and to other professional communities that have not yet developed similar processes, as it demonstrates a functional model that can be adapted elsewhere.

The volunteer database has already been used to match candidates with requests from AAPM committees, such as identifying volunteers with specialized skill sets or language capabilities and translating educational materials into multiple languages, demonstrating its immediate value in facilitating real‐world connections. Thus, this model complements, rather than replaces, traditional in person training programs, offering a cost‐effective and sustainable strategy to expand the global reach of medical physics expertise.

In addition to the volunteer database, the RadCollab project intake database has been developed to complement the volunteer system by providing a centralized platform for international project coordination. While the volunteer database matches AAPM members with global opportunities based on their expertise and availability, RadCollab streamlines the intake and management of global medical physics projects, ensuring collaboration is efficient and efforts are not duplicated.

The integration of these two databases creates a powerful synergy: the volunteer database supplies the expertise, while RadCollab offers the infrastructure for project management and data sharing. Together, they will enhance global collaborations in radiation medicine, facilitating the seamless connection of qualified volunteers with international projects and amplifying the impact of both initiatives. This holistic approach strengthens AAPM's mission to address healthcare disparities and expand access to advanced medical physics technologies worldwide.

This study has several limitations, the most important being the low response rate. Only about 4% of AAPM members (427 responses) completed at least one questionnaire, which limits the generalizability of the findings. Although this sample size gives a margin of error of about 5% at the 95% confidence level, the bigger concern is non‐response bias, since respondents may not represent the overall membership demographics. As such, the results should be interpreted as exploratory and hypothesis‐generating rather than predictive of the entire AAPM membership. Another limitation of this study is the absence of demographic information about the volunteers, and this may include a potential biasing to the presented results.

The current volunteer database includes expertise spanning external beam radiotherapy, brachytherapy, imaging, quality assurance, and education/training, which aligns well with many of the needs commonly identified by LMIC institutions. This breadth provides a solid foundation for meaningful engagement; however, certain areas of expertise remain underrepresented, including mentoring in specialized clinical skills and platform‐specific experience. Addressing these gaps through targeted recruitment and collaboration will be important to ensure comprehensive support. Overall, the current volunteer base is well positioned to address a substantial portion of LMIC needs as an initial step, while also highlighting clear priorities for future expansion.

Future work will focus on ongoing maintenance to ensure the accuracy and reliability of volunteer information over time, as well as enabling broader access to search functionality. To expand the database, we will encourage new applications and promote quarterly updates of existing profiles through periodic reminder and targeted chapter outreach. Additional improvements may include the development of analytics tools to assess system utilization and identify factors contributing to underutilization. Using these tools, success will be measured through both quantitative metrics, such as the number of active volunteers and the frequency of successful matches, and qualitative metrics, including user satisfaction questionnaires. Ultimately, we aim to promote the sustainability and effectiveness of the platform for meaningful global collaboration in medical physics.

## 5 CONCLUSION

This work presents the creation of a comprehensive volunteer database, aimed at collecting the expertise of AAPM member volunteers and simplifying the process of matching the specific training needs with the expertise of AAPM volunteers. The study results provide insights into the active engagement of AAPM members in international initiatives, current availability of diverse expertise, and collaborations with various organizations in the field of medical physics. It underscores the broad range of expertise and willingness of AAPM members to contribute to education across all specialties, advancing global medical physics. This project lays the foundation for a sustainable framework to leverage AAPM members’ skills and knowledge to improve global access to high quality healthcare, aid the advancement of the practice of medical physics globally, and help to address disparities in healthcare.

## AUTHOR CONTRIBUTIONS

TL drafted the initial draft, MK, CM, JZ, KL, and PS contributed to the editing of the draft, YX and MW facilitated the data collection, EH finalized the draft and oversaw the project.

## CONFLICT OF INTEREST STATEMENT

The authors have no conflict of interest to disclose.

## Supporting information



Supporting information
