# Supplementary material for: Design and implementation of an AAPM volunteer database for advancing global medical physics initiatives
Source: J Appl Clin Med Phys. 2026 Feb 19;27(2):e70511. doi: 10.1002/acm2.70511 (PMC12919486; doi:10.1002/acm2.70511)
Supplement: Supplementary file 1 — Supporting information [file ACM2-27-e70511-s001.pdf]

AAPM Member Profile GDIEC Diagnostic Questions

The goal of the recently established International Council (IC) of the American Association of Physicists in Medicine (AAPM) is to identify and develop strategies for advancing the practice of medical physics globally, address disparities in healthcare and develop mitigation strategies in collaboration with other global stakeholders that include international medical physics organizations and non-profit-organizations. To maximize the impact of the IC's work there is a need to create and maintain a cadre of volunteers within the AAPM membership who would be willing and able to volunteer their time and effort towards various International Council activities for collaborating with institutions and partners in different world regions to address these needs. The Global Data and Information Exchange Committee has added additional optional data elements for the AAPM member profile to allow members interested in participating in IC volunteer activities to maintain their volunteer interests.

Your input will only be visible to AAPM IC committee members. Once this information is compiled, the committee members may reach out to you through the email listed in your member profile.

We appreciate your help and interest in the mission of the AAPM and the International Council.

Click here if you wish to remove yourself from consideration.

Expertise with Diagnostic Machine Types

Radiography
Check all that apply.

- [ ] Screen-film
- [ ] Digital radiography (CR and DR)
- [ ] Dual-energy x-ray absorptiometry

Other: ________

Select All

Mammography
Check all that apply.

- [ ] Screen-film
- [ ] Full-field digital mammography
- [ ] Digital breast tomosynthesis
- [ ] Contrast-enhanced mammography
- [ ] Stereotactic breast biopsy

Other: ________

Select All

Fluoroscopy/Interventional radiography
Check all that apply.

- [ ] Mobile C-arms
- [ ] Interventional C-arms
- [ ] Radiographic/fluoroscopic systems

Other: ________

Select All

Computed tomography
Check all that apply.

- [ ] GE
- [ ] Philips
- [ ] Siemens

- [ ] Canon/Toshiba
- [ ] Fujifilm/Hitachi
- [ ] United Imaging
- [ ] Dual-energy/multi-energy CT

Other: ______

Select All

Magnetic resonance imaging

Check all that apply.

- [ ] GE
- [ ] Philips
- [ ] Siemens
- [ ] Canon/Toshiba
- [ ] Fujifilm/Hitachi
- [ ] United Imaging
- [ ] Hyperfine

Other: ______

Select All

Ultrasound

Check all that apply.

- [ ] Obstetric and Gynecologic Sonography
- [ ] Cardiovascular and Vascular Sonography
- [ ] Breast Sonography
- [ ] Abdominal Sonography
- [ ] Musculoskeletal Sonography
- [ ] Pediatric Sonography

Other: ______

Select All

Expertise with Software

- [ ] PACS Configuration and management - Enterprise Imaging
- [ ] Dose monitoring software
- [ ] Radiology Information Systems

Specialty Areas of Interest of clinical training/teaching

Enter Specialty Areas of Interest

Check all that apply.

- [ ] Radiography
- [ ] Mammography
- [ ] Fluoroscopy
- [ ] CT
- [ ] MRI
- [ ] US
- [ ] Informatics

- [ ] Machine learning
- [ ] Digital phantoms
- [ ] Physical phantoms
- [ ] Quantitative imaging
- [ ] Radiomics
- [ ] Quality control

Other: ____

Select All

How would you like to participate?

Which International Council Programs are you interested in volunteering?
Check all that apply.

- [ ] Clinical Education Training Program (GCETC)
- [ ] Medical Physics Education and Training Program (GMPETC)
- [ ] Global Needs and Assesment Program (GNAC)

Other: ____

Select All

If you have any regional/cultural preference, what are they?
Check all that apply.

- [ ] North America
- [ ] South America
- [ ] Asia
- [ ] Middle East
- [ ] Africa
- [ ] Europe
- [ ] Oceania

Please add countries if you desired to specify: ____

Select All

Funding requirement for on-site visits
Mark only one.

- ○ I don't have funding to cover any expenses.
- ○ If flights are covered by AAPM or the host hospital, I can cover other expenses
- ○ I have funding to cover all expenses.
- ○ Other: ____

Involvement of international activities

Are you already involved in international activities? If so, please tell us how:

Involvement through other AAPM Programs: (please specify)

____

Involvement with other non-profits/non-governmental organization: (please specify)

____

Involvement through Institutional efforts: (please specify)

____

Involvement through personal connections:(please specify)

Other

Teaching seminar/workshop/courses

Visiting preferences

Mark only one.

- ◯ In-person visit preferred.
- ◯ On-line visit preferred.
- ◯ No preference
- ◯ Other:

Could you provide recording of the lectures?

Mark only one.

- ◯ Yes
- ◯ No

How long could you volunteer per each (in person or virtual) visit?

Mark only one.

- ◯ < 1 week
- ◯ < 1 month
- ◯ < 1 year
- ◯ > 1 year
- ◯ Occasional lectures
- ◯ Other:

Submit

If you have questions or concerns, please reach out to the committee at **2025.gdiec@aapm.org**

AAPM Member Profile GDIEC Nuclear Medicine Questions

The goal of the recently established International Council (IC) of the American Association of Physicists in Medicine (AAPM) is to identify and develop strategies for advancing the practice of medical physics globally, address disparities in healthcare and develop mitigation strategies in collaboration with other global stakeholders that include international medical physics organizations and non-profit-organizations. To maximize the impact of the IC's work there is a need to create and maintain a cadre of volunteers within the AAPM membership who would be willing and able to volunteer their time and effort towards various International Council activities for collaborating with institutions and partners in different world regions to address these needs. The Global Data and Information Exchange Committee has added additional optional data elements for the AAPM member profile to allow members interested in participating in IC volunteer activities to maintain their volunteer interests.

Your input will only be visible to AAPM IC committee members. Once this information is compiled, the committee members may reach out to you through the email listed in your member profile.

We appreciate your help and interest in the mission of the AAPM and the International Council.

Click here if you wish to remove yourself from consideration.

Expertise with Nuclear Medicine Instrumentation

Check all that apply.

- [ ] PET
- [ ] SPECT
- [ ] SPECT/CT hybrid imaging
- [ ] PET/CT hybrid imaging
- [ ] PET/MR hybrid imaging
- [ ] Gamma camera
- [ ] Image acquisition hardware
- [ ] Preclinical imaging
- [ ] Non-imaging nuclear hardware

Other: ____

Select All

Specialty Areas of Interest of clinical training/teaching

Check all that apply.

- [ ] Image reconstruction and processing
- [ ] Radionuclide therapy
- [ ] Cyclotron physics and radiopharmaceutical production
- [ ] Quantitative imaging
- [ ] Kinetic Modeling
- [ ] Quality control and image quality assessment
- [ ] Radiation dosimetry & radiation safety

Other: ____

Select All

How would you like to participate?

Which International Council Programs are you interested in volunteering?
Check all that apply.

- [ ] Clinical Education Training Program (GCETC)

- [ ] [Medical Physics Education and Training Program (GMPETC)]
- [ ] [Global Needs and Assesment Program (GNAC)]

Other:

Select All

If you have any regional/cultural preference, what are they?
Check all that apply.

- [ ] North America
- [ ] South America
- [ ] Asia
- [ ] Middle East
- [ ] Africa
- [ ] Europe
- [ ] Oceania

Please add countries if you desired to specify:

Select All

Funding requirement for on-site visits
Mark only one.

- ( ) I don't have funding to cover any expenses.
- ( ) If flights are covered by AAPM or the host hospital, I can cover other expenses
- ( ) I have funding to cover all expenses.
- ( ) Other:

Involvement of international activities

Are you already involved in international activities? If so, please tell us how:

Involvement through other AAPM Programs: (please specify)

Involvement with other non-profits/non-governmental organization: (please specify)

Involvement through Institutional efforts: (please specify)

Involvement through personal connections:(please specify)

Other

Teaching seminar/workshop/courses

Visiting preferences
Mark only one.

- ( ) In-person visit preferred.
- ( ) On-line visit preferred.
- ( ) No preference
- ( ) Other:

Could you provide recording of the lectures?

Mark only one.

- ◯ Yes
- ◯ No

How long could you volunteer per each (in person or virtual) visit?

Mark only one.

- ◯ < 1 week
- ◯ < 1 month
- ◯ < 1 year
- ◯ > 1 year
- ◯ Occasional lectures
- ◯ Other: ____________

Submit

AAPM Member Profile GDIEC Therapy Questions

The goal of the recently established International Council (IC) of the American Association of Physicists in Medicine (AAPM) is to identify and develop strategies for advancing the practice of medical physics globally, address disparities in healthcare and develop mitigation strategies in collaboration with other global stakeholders that include international medical physics organizations and non-profit-organizations. To maximize the impact of the IC's work there is a need to create and maintain a cadre of volunteers within the AAPM membership who would be willing and able to volunteer their time and effort towards various International Council activities for collaborating with institutions and partners in different world regions to address these needs. The Global Data and Information Exchange Committee has added additional optional data elements for the AAPM member profile to allow members interested in participating in IC volunteer activities to maintain their volunteer interests.

Your input will only be visible to AAPM IC committee members. Once this information is compiled, the committee members may reach out to you through the email listed in your member profile.

We appreciate your help and interest in the mission of the AAPM and the International Council.

Click here if you wish to remove yourself from consideration.

Expertise with Radiotherapy Machines and TPS

External beam treatment machines
Check all that apply.

- ☐ C-arm (TrueBeam, etc.) - Varian
- ☐ C-arm (Versa HD, etc.) - Elekta
- ☐ Tomotherapy (Accuray)
- ☐ Ethos, Halcyon (Varian)
- ☐ Unity (Elekta)
- ☐ Mridian (ViewRay)
- ☐ Gamma Knife
- ☐ Cyberknife
- ☐ RefleXion

Other: ____________

Select All

Treatment Planning System
Check all that apply.

- ☐ Eclipse
- ☐ RayStation
- ☐ Pinnacle
- ☐ Monaco

Other: ____________

Select All

HDR Brachytherapy
Check all that apply.

- ☐ Bravos, Varisource, GammaMed - Varian
- ☐ Flexitron, MicroSelectron - Elekta
- ☐ Co-60 Afterloader

Other: ____________

Select All

LDR brachytherapy
Check all that apply.

- [ ] Prostate
- [ ] Eye plaque
- [ ] GYN intracavitary

Other:

Select All

Proton / particle therapy
Check all that apply.

- [ ] Pencil beam scanning
- [ ] Double scattering

Other:

Select All

Intraoperative radiotherapy (IORT)
Check all that apply.

- [ ] Electron (Mobetron, etc.)
- [ ] X-rays (Intrabeam, etc.)

Other:

Select All

CT Simulator
Check all that apply.

- [ ] GE
- [ ] Phillips
- [ ] Siemens

Other:

Select All

Radiation Oncology EMR
Check all that apply.

- [ ] Radiation Oncology EMR Configuration and administration - ARIA
- [ ] Radiation Oncology EMR Configuration and administration - MOSAIQ

Other:

Select All

Specialty Areas of Interest of clinical training/teaching

Enter Specialty Areas of Interest
Check all that apply.

- [ ] Image registration techniques
- [ ] Image segmentation techniques
- [ ] 3-Dimensional Conformal Radiation Therapy (3DCRT)
- [ ] Intensity modulated radiation therapy (IMRT)
- [ ] Volumatic Modulated Arc Therapy (VMAT)

- [ ] Image-guided radiation therapy (IGRT)
- [ ] Stereotactic body radiation therapy (SBRT)
- [ ] Stereotactic Radiosurgery (SRS)
- [ ] Total Skin Electron Therapy (TSET)
- [ ] Total Body Irradiation (TBI)
- [ ] Motion management
- [ ] Treatment planning
- [ ] Radiation dose measurement and calibration
- [ ] Machine and patient quality assurance
- [ ] Treatment outcome modeling
- [ ] Non-ionizing radiation therapies

Other:

Select All

How would you like to participate?

Which International Council Programs are you interested in volunteering?
Check all that apply.

- [ ] Clinical Education Training Program (GCETC)
- [ ] Medical Physics Education and Training Program (GMPETC)
- [ ] Global Needs and Assesment Program (GNAC)

Other:

Select All

If you have any regional/cultural preference, what are they?
Check all that apply.

- [ ] North America
- [ ] South America
- [ ] Asia
- [ ] Middle East
- [ ] Africa
- [ ] Europe
- [ ] Oceania

Please add countries if you desired to specify:

Select All

Funding requirement for on-site visits
Mark only one.

- ○ I don't have funding to cover any expenses.
- ○ If flights are covered by AAPM or the host hospital, I can cover other expenses
- ○ I have funding to cover all expenses.
- ◉ Other:

Involvement of international activities

Are you already involved in international activities? If so, please tell us how:

Involvement through other AAPM Programs: (please specify)

Involvement with other non-profits/non-governmental organization: (please specify)

Involvement through Institutional efforts: (please specify)

Involvement through personal connections:(please specify)

Other

Teaching seminar/workshop/courses

Visiting preferences

Mark only one.

- ○ In-person visit preferred.
- ○ On-line visit preferred.
- ○ No preference
- ◉ Other:

Could you provide recording of the lectures?

Mark only one.

- ◉ Yes
- ○ No

How long could you volunteer per each (in person or virtual) visit?

Mark only one.

- ○ < 1 week
- ○ < 1 month
- ○ < 1 year
- ○ > 1 year
- ○ Occasional lectures
- ◉ Other:

Submit

If you have questions or concerns, please reach out to the committee at 2025.gdiec@aapm.org
